# Effect of Montelukast sodium combined with Budesonide aerosol on airway function and T lymphocytes in asthmatic children

**DOI:** 10.12669/pjms.38.5.5749

**Published:** 2022

**Authors:** Wei Jin, Zichong Zhao, Dongping Zhou

**Affiliations:** 1 Wei Jin, Department of Paediatrics, Huzhou Maternity & Child Health Care Hospital, Huzhou 313000, Zhejiang Province, P.R. China; 2 Zichong Zhao, Department of Paediatrics, Huzhou Maternity & Child Health Care Hospital, Huzhou 313000, Zhejiang Province, P.R. China; 3 Dongping Zhou Department of Paediatrics, Huzhou Traditional Chinese Medicine Hospital Affiliated to Zhejiang Chinese Medical University, Huzhou 313000, Zhejiang Province, P.R. China

**Keywords:** Asthma in children, Montelukast sodium, Budesonide atomizer, T -lymphocytes, Airway function

## Abstract

**Objectives:**

To investigate the effects of Montelukast sodium combined with Budesonide aerosol on airway function and T lymphocytes in asthmatic children.

**Methods:**

The records of 86 pediatric asthma patients, treated in Huzhou Maternal and Child Health Hospital from February 2020 to March 2021, were studied retrospectively. Of them, 40 children received routine treatment + budesonide atomizer (Group-I), and 46 patients received routine treatment + budesonide atomizer + montelukast sodium (Group-II). The improvement in airway and lung function, and T-lymphocyte count in both groups after 3 months of corresponding treatment were analyzed.

**Results:**

After three months of treatment, expiratory flow rate (TEF) with the tidal volume of 25%, 50% and 75%, was significantly higher in Group-II than Group-I (P<0.05). CD8+ expression in Group-II was lower, and CD3+, CD4+ and CD4+/CD8+ were higher than those in Group-I (P<0.05). There was a significant difference in the levels of inflammatory factors between the two groups. The levels of IL-4, IL-5 and IFN-γ in Group-II were lower than those in Group-I(P<0.05).

**Conclusions:**

In the clinical treatment of asthmatic children, in combination with routine treatment, budesonide atomizer and montelukast sodium can effectively promote the improvement of airway function, regulate T lymphocytes levels, reduce inflammatory reaction and improve the total clinical curative effect.

## INTRODUCTION

Asthma is a common chronic respiratory disease. In children, pathological changes are mainly chronic airway inflammation and airway hyperresponsiveness, and the clinical symptoms are mainly chest tightness, cough, shortness of breath, wheezing, etc.^1^ If the condition of children with asthma is not effectively controlled on time, it can have a serious impact on their physical and mental health, normal growth and development, and even endanger their life.^2^ At present, routine treatment involves controlling phlegm, relieving cough and reducing inflammation and infection. Budesonide is an inhaled glucocorticoid commonly used in the clinical treatment of pediatric asthma. It has good anti-inflammatory effect and positive curative effect. However, previous studies and clinical practice show that budesonide has relatively little inhibitory effect on leukotrienes during application, and its overall efficacy still needs to be improved.^3^ Montelukast sodium is a leukotriene receptor antagonist and is also widely used in the clinical treatment of asthma. Montelukast sodium can effectively inhibit the activity of leukotriene polypeptide in airway smooth muscle, thus efficiently reversing increased vascular permeability. Montelukast sodium treatment was shown to alleviate bronchospasm, inhibit eosinophil infiltration, help reduce airway hyperresponsiveness and promote the improvement of lung function.^4^ It is reported that the simultaneous use of budesonide and montelukast sodium may have synergistic effect, make up for the shortcomings of budesonide alone, and more effectively improve the immune function of patients.^5^

This study discusses in depth the effect and value of combined budesonide and montelukast sodium administration in the clinical treatment of children with asthma, to provide more valuable reference for the further optimization of the treatment scheme of children with asthma.

## METHODS

Eighty six children with asthma were treated in pediatric department of Huzhou Maternal and Child Health Hospital from February 2020 to March 2021.

### Inclusion criteria:

- Complete relevant clinical data and medical records of diagnosis and treatment;
- Meeting diagnostic criteria of childhood asthma within the guidelines for the diagnosis, prevention and treatment of childhood bronchial asthma,^6^ and confirmed by relevant examination and symptom observation;
- Age≤12 years;
- Good compliance during diagnosis and treatment, and no history of allergy to the drugs used in the study.

### Exclusion criteria:

- The disease in acute attack stage;
- Cough caused by other reasons or accompanied by congenital heart disease, pulmonary diseases and other respiratory diseases;
- Cognitive or language dysfunction and mental illness.

All processes of this study fully comply with the relevant rules and regulations of the medical ethics committee of Huzhou Maternal and Child Health Hospital (Approval number: HZFY-L21041701, Date: 2021-04-17), and informed consent was obtained from all patients.

According to the different drugs used in the treatment of children, the children were divided into routine treatment and budesonide atomizer treatment group (Group-I) and routine treatment, budesonide atomizer and montelukast sodium treatment group (Group-II). The number of cases in the two groups were 40 and 46 respectively.

Treatment plan of Group-I: routine treatment that included cough and asthma relief, oxygen inhalation anti infection treatment and oxygen inhalation treatment immediately after admission, while maintaining the balance of acid-base, water and electrolyte in the children, intravenous drip of terbutaline sulfate injection, and then atomized inhalation of budesonide atomizer. The drug dose for children aged ≥7 years was 500 μg/time, 300 for children<7 years old μg/time, 3times/d; Group-II took montelukast sodium 4 mg once a day on the basis of the treatment of Group-I.

The changes in airway function [expiratory flow rate (TEF) at 25%, 50% and 75% tidal volume measured by master scope pulmonary function tester] before and after treatment were recorded and compared between the two groups. Ten milliliters of peripheral venous blood were taken before and after treatment, and the T- lymphocyte count in both groups was compared; CD3+, CD4+, CD8+ were measured by FACScalibur flow cytometry, and the changes in CD4 + / CD8 + were calculated. Levels of serum inflammatory factors γ- interferon (IFN-γ), interleukin-4 (IL-4) and interleukin-5 (IL-5) were measured by enzyme-linked immunosorbent assay.

### Statistical Analysis:

SPSS 22.0 software was used for statistical analysis. The counting data are expressed as (x̄ ±s); One way ANOVA was used for comparison between groups; Continuous data are expressed in percentage n[%]; Comparison between groups was done using χ^2^ statistics. P<0.05 indicates that the difference is statistically significant, and the test level is high α=0.05.

## RESULTS

There was no significant difference in before-treatment TEF between the two groups when the tidal volume was 25%, 50% and 75% (P > 0.05). After three months of treatment with corresponding drugs, the TEF of children in both groups was significantly higher than that before treatment (P<0.05) (Table-I). After-treatment TEF of patients in Group-II (routine treatment, budesonide atomizer and montelukast sodium) was higher than that in Group-I, and the difference was statistically significant (P<0.05) (Table-II).

**Table I:** Comparison of basic data between the two groups.

Group	n	Sex ratio	Age (year,χ¯ ±s)	Course of disease (d, χ¯ ±s)

		(Male*/Female*)		
Group-I	40	24/16	4~12(7.75±2.19)	3~14(7.95±2.97)
Group-II	46	26/20	3~12(7.84±2.26)	3~15(8.26±2.99)
*χ^2^*/*t*	-	0.106	0.203	0.482
*P*	-	0.744	0.840	0.631

**Table II:** Comparison of TEF measurement results between the two groups before and after treatment (χ¯±s).

Group	n	TEF 25%(mL/s)		TEF 50%(mL/s)		TEF 75%(mL/s)	

		Before treatment	After treatment	Before treatment	After treatment	Before treatment	After treatment
Group-I	40	76.62±4.11	81.42±4.09	105.75±7.43	113.52±7.46	123.92±6.43	130.07±6.07
Group-II	46	76.15±3.48	87.02±3.68	106.89±7.31	125.20±6.54	124.41±5.05	153.87±4.96
*t*		0.578	6.677	0.717	7.731	0.394	20.007
*P*		0.565	*P*<0.001	0.476	*P*<0.001	0..695	*P*<0.001

Three months after the corresponding treatment, the levels of CD3+, CD4+ and CD4+/CD8+ in both groups were significantly higher than before treatment, and the levels of CD8+ were significantly lower than those before treatment (P<0.05). At the same time, after treatment, compared with Group-I, children in Group-II had significantly higher levels of CD3+, CD4+, CD4+/CD8+ and lower CD8+ (P<0.05) (Table-III).

**Table III:** Comparison of T lymphocyte test results between the two groups before and after treatment.

Group	Time	CD3+(%)	CD4+(%)	CD8+(%)	CD4+/CD8+
Group-I	Before treatment	55.37±3.01	33.37±3.70	31.77±3.78	1.05±0.03
Group-II		54.78±3.62	33.19±3.37	31.93±3.27	1.04±0.04
Inspection value	*t*	0.828	0.235	0.223	1.236
	*P*	0.410	0.814	0.824	0.220
Group-I	After treatment	58.55±2.93	36.30±3.67	28.00±3.22	1.29±0.06
Group-II		61.84±3.49	41.22±3.36	23.93±2.99	1.73±0.13
Inspection value	*t*	4.779	6.489	6.052	19.196
	*P*	*P<*0.001	*P<*0.001	*P<*0.001	*P<*0.001

Before treatment, there was no significant difference in the levels of related inflammatory factors between the two groups (P > 0.05). After three courses of treatment, the levels of IFN-γ, IL-4 and IL-5 in the two groups were significantly lower than those before treatment (P<0.05). After treatment, the levels of blood IL-4, IL-5 and IFN-γ in Group-II were significantly lower than those in Group-I (P<0.05) (Table-IV).

**Table IV:** IFN before and after treatment- γ and IL-4 and IL-5 levels (χ¯±s).

Group	n	IFN-γ(ng·L^-1^)		IL-4(ng·L^-1^)		IL-5(ng·L^-1^)	

		Before treatment	After treatment	Before treatment	After treatment	Before treatment	After treatment
Group-I	40	589.00±18.35	284.22±15.04	274.92±10.11	235.75±9.57	222.92±12.23	123.80±12.29
Group-II	46	591.30±20.13	218.78±21.41	276.06±9.86	122.95±12.92	224.56±12.69	96.04±12.77
*t*	-	0.551	16.559	0.529	46.355	0.608	10.227
*P*	-	0.585	*P*<0.001	0.598	*P*<0.001	0.545	*P*<0.001

## DISCUSSION

This study demonstrated that a combination of budesonide aerosol and montelukast sodium treatment significantly improves airway function in children with asthma, has anti-inflammatory effect, promotes better regulation of T lymphocyte levels, and improves the overall clinical effect of the treatment.

Childhood asthma is a heterogeneous disease characterized by chronic airway inflammation, and is related to many factors, such as heredity, infection, endocrine disorders, food allergies, environment, autoimmune abnormalities and so on. Typical symptoms of asthma in children are chest tightness, shortness of breath, wheezing, cough and other respiratory manifestations, accompanied by varying degrees of variable expiratory airflow restriction. The severity of symptoms may change over time. Asthma can occur in any age group, with children aged 4 ~ 5 comprising highest-incidence group. Clinical data show that the incidence of asthma in children has increased over the years, and the prevalence in China is currently 0.5% to 2%.^7^ Asthma not only has a serious impact on children’s respiratory tract but may also lead to a series of complications such as emphysema, rib fracture, thoracic deformity and cor pulmonale with the aggravation of the disease, which affects overall health, normal growth and development, and reduce the quality of life. Therefore, at present, pediatric asthma has become one of the important diseases that seriously affect children’s health and growth. Active prevention and treatment of pediatric asthma has become a hot topic.

In asthma, airway function is significantly reduced, and the related inflammatory cells release a large amount of inflammatory transmitter leukotriene, which, in combination with other inflammatory factors, can aggravate the airway inflammation of patients.^8^ Since asthma is a chronic airway persistent inflammatory reaction caused by immunological abnormalities, accompanied by airway hyperresponsiveness,^9^ treatment mechanisms mainly focus on the improvement of airway function, control of inflammatory response and the improvement of immune function. Timely selection of appropriate drug therapy at the early stages of the disease are crucial for the successful improvement of symptoms and prevention of possible complications.

Budesonide is an adrenocortical drug that has high affinity to adrenocortical hormone receptor, and can interfere with the activation and chemotaxis of eosinophils, and effectively inhibits secretion and synthesis of inflammatory factors, thus having an overall local anti-inflammatory effect.^10^ Studies have also demonstrated that budesonide functions as immunoregulator and inhibitor of bronchial smooth muscle contraction.^11^ At present, budesonide is widely used in the clinical treatment of infant respiratory diseases, with good results. Budesonide aerosol inhalation for asthmatic children can effectively inhibit local IgE production in the airway, promote the reduction of IgE activity, improve the capillary permeability and inhibit the release of allergic media.^12^ At the same time, this kind of drug atomization inhalation can also prevent edema by contracting capillaries, inhibit the transfer of inflammatory cells to the site of inflammation attack, and thus prevent the occurrence or aggravation of airway inflammation in children.^13^ Ismail G et al.^14^ showed that budesonide can effectively inhibit the redistribution of eosinophils and T lymphocytes in blood circulation, and plays an anti-inflammatory and immune function improvement role. Using budesonide by atomization inhalation can obtain more ideal lung absorption effect, improve drug concentration, ensure drug efficacy, reduce drug entry into blood circulation and reduce adverse drug reactions, especially for asthmatic children with relatively poor tolerance. Chen J et al.^15^ showed that clinical symptoms and pulmonary function of patients were significantly improved after treatment with budesonide. However, clinical studies show that budesonide alone cannot significantly inhibit all inflammatory factors, especially leukotriene. The effect of inflammation control and improvement of airway function are limited to a certain extent, and long-term use of glucocorticoids can lead to adverse reactions such as gastrointestinal reaction, abnormal blood glucose and myofibrosis. Therefore, there is still a need to further improve and optimize clinical treatment of pediatric asthma.

Montelukast sodium is a widely used leukotriene receptor antagonist, which can effectively inhibit the release of leukotriene, reduce its activity, and subsequently reduce leukotriene-mediated inflammatory response. Montelukast sodium can also effectively promote tracheal dilation, significantly alleviate the contraction of suction smooth muscle, and improve airway hyperresponsiveness. Glockler Lauf SD et al.^16^ pointed out that the application of montelukast sodium can significantly reduce blood eosinophils, significantly inhibit airway remodeling and prevent anti-pulmonary fibrosis. According to the research results of Luo H et al.,^17^ montelukast sodium treatment significantly improved levels of T lymphocytes in children with asthma. Moreover, studies show that montelukast sodium can effectively inhibit and prevent peptide growth factor, reduce or prevent airway eosinophil infiltration, reduce bronchospasm in asthmatic children and control airway inflammatory response. Some studies have shown that after the traditional anti-inflammatory treatment is given to asthmatic children, administration of montelukast sodium significantly improved asthmatic symptoms and reduced levels of inflammatory factors. However, montelukast sodium treatment had no significant effect on the overall course of the disease.^18^ Application of montelukast sodium in the clinical treatment of pediatric asthma can effectively make up for the shortcomings of budesonide. When combined, budesonide and montelukast sodium may have a synergistic effect, thus potentially improving clinical manifestations of the disease in pediatric patients.

Zhang Y et al.^19^ showed that compared with budesonide alone, the treatment effect of budesonide+ montelukast sodium regimen for asthmatic children is more comprehensive, and the improvement effects of children’s clinical symptoms, airway function and lung function are more significant. The results of this study on 86 asthmatic children showed that after the children in Group-II received budesonide atomization inhalation and oral montelukast sodium on the basis of routine treatment, the TEF of 25%, 50% and 75% in this group were significantly higher than those in Group-I. Levels of CD3+, CD4+, CD4+/CD8+ were higher, while CD8+ was lower in children, receiving a combined treatment. Among the serum inflammatory factors, IL-4, IL-5 and IFN-γ were significantly lower in Group-II patients, and the difference between the groups was statistically significant. Our results demonstrate that the combination of budesonide and montelukast sodium in the treatment of asthmatic children can significantly improve the effect of therapy on airway function, effectively reduce inflammatory reaction and promote better regulation of T lymphocytes in children. TEF is an important marker of severe airway spasm in children with asthma when the tidal volume is 25%, 50% and 75%. The combined use of the two drugs can improve the control effect of chronic inflammation and significantly reduce the airway hyper responsiveness in children. T lymphocytes can directly reflect the cellular immune status of the body. The combined use of two drugs can greatly affect the activation of T lymphocytes in asthmatic children and play a regulatory role in immune function.

### Limitations of the study:

The current study is retrospective in nature, and the sample size is relatively small. Longer follow-up studies with larger sample sizes are needed to investigate the effect of different treatment regiments on pediatric asthma patients.

## CONCLUSION

The combination of budesonide aerosol and montelukast sodium in the clinical treatment of asthmatic children can significantly improve the airway function, has marked anti-inflammatory effect, allowed better regulation of T lymphocyte levels, and improves the overall clinical effect.

### Authors’ contributions:

**WJ:** Conceived and designed the study.

**ZZ & DZ:** Collected the data and performed the analysis.

**WJ:** Was involved in the writing of the manuscript and tis responsible for clinical integrity of the study. All authors have read and approved the final manuscript.
